# Solitary Nanostructures Produced by Ultrashort Laser Pulse

**DOI:** 10.1186/s11671-016-1381-1

**Published:** 2016-04-05

**Authors:** Nail A. Inogamov, Vasily V. Zhakhovsky, Viktor A. Khokhlov, Yury V. Petrov, Kirill P. Migdal

**Affiliations:** Landau Institute for Theoretical Physics, Russian Academy of Sciences, Chernogolovka, Russia; Dukhov Research Institute of Automatics, ROSATOM, Moscow, Russia; Moscow Institute of Physics and Technology (State University), Dolgoprudny, Russia

**Keywords:** Ultrashort laser pulse, Thin film on substrate, Formation of microbump, Molecular dynamics, **PACS Codes**, 79.20.Eb, 62.50.Ef, 02.70.Ns

## Abstract

Laser-produced surface nanostructures show considerable promise for many applications while fundamental questions concerning the corresponding mechanisms of structuring are still debated. Here, we present a simple physical model describing those mechanisms happened in a thin metal film on dielectric substrate irradiated by a tightly focused ultrashort laser pulse. The main ingredients included into the model are (i) the film–substrate hydrodynamic interaction, melting and separation of the film from substrate with velocity increasing with increase of absorbed fluence; (ii) the capillary forces decelerating expansion of the expanding flying film; and (iii) rapid freezing into a solid state if the rate of solidification is comparable or larger than hydrodynamic velocities. The developed model and performed simulations explain appearance of microbump inside the focal spot on the film surface. The model follows experimental findings about gradual transformation of the bump from small parabolic to a conical shape and to the bump with a jet on its tip with increasing fluence. Disruption of the bump as a result of thinning down the liquid film to a few interatomic distances or due to mechanical break-off of solid film is described together with the jetting and formation of one or many droplets. Developed theory opens door for optimizing laser parameters for intended nanostructuring in applications.

## Background

Fabrication of surface structures and/or nanodroplets is a popular direction in very promising laser applications. Such structures are used for surface-enhanced Raman spectroscopy (SERS) and tip-enhanced Raman spectroscopy (TERS) [1]. The enhancement of a Raman signal is based on the following. (i) The sub-wavelength structure/tip, like a frozen jet above the top of a bump, is produced. (ii) The structure is illuminated by weak light. (iii) There are spots of sub-wavelength volume (hot-spots) near the tip where orders of magnitude enhancement of a weak illuminating electromagnetic field takes place. (iv) The Raman effect is approximately proportional to the fourth power of the field. Thus, the Raman signal from a single molecule caught into the hot-spot can be enhanced up to the detectable level. In the similar way, using nanostructures and hot-spots, the photoluminescence from a single molecule under illumination by weak light is measured [2]. In another limit of strong illuminating laser pulse, the surface nanostructures amplify (perhaps again as a result of the hot spot enhancement) electron and X-ray emission from the surface [3].

Two–four ray interference of an ultrashort pulse is applied to produce a two-dimensional lattice of solitary bumps or holes on a thin film [4, 5]. Using UV laser pulse (second harmonics of femtosecond laser 785 nm), the lattice spacing between neighboring nanobumps on the thin gold film may be done less than a micrometer (spatial period of a lattice is 760 nm in [6]); exactly this type of bumps are considered in this paper. These plane two-dimensional “crystals” enhance absorption of light by dye [7]. This is important for the super sensor technique like the surface-enhanced infrared absorption (SEIRA) solving similar problems as SERS. The grating array is a fundamental component in nanophotonics. It can include metamaterials which show a negative refractive index and harmonic conversion [5, 8].

The bumps and tips are used in nanophotonics/plasmonics as antennas for operations with light [1, 9]. The bumps or solitary undersurface frozen bubbles can be utilized for production of two-dimensional photon crystals. Such bubbles were found and studied in [10–12]. Surface nanostructures are also employed for colorizing, tribology, and manipulations with wettability [13–15] as well as in bio-applications [16].

Another mainstream of laser-assisted applications is the production of nanoparticles for different technologies [17–21]: fabrication of nanoparticles for plasmonic applications [18, 19], laser-induced forward/backward transfer (LIFT, LIBT) [17], pulsed laser deposition (PLD) [20], femtosecond laser production of nanoparticles through thin film ablation (fs-TTFA) [21].

Thermomechanical separation of film from substrate described below is a real reason for the bump formation. Higher absorbed energy *F*_*c*_ in Eq. (1) results in jetting from the top of bump. By increasing *F*_*c*_ either a part of the jet, or whole jet, or whole jet together with a part of bump disintegrates into flying droplets. Thus, the production of droplets can gradually change from a single droplet to a few and then many droplets with increasing *F*_*c*_.

Droplets are characterized by distributions *dn*/*dm,dn*/*dE,dn*/*dΩ* throughout mass, kinetic energy, and an angle of escape. The simulations described below show how to adjust, e.g., the flying angle of droplet or to regulate the number of droplets. This is possible in the case of solitary nanobump with small size *R*_*L*_∼1 *μ*m in (1). For larger bump size, the conditions are less controllable. New observation given in the paper concerns the appearance of *solid* debris in an ejecta cloud; see Fig. 7 below. Their presence is caused by inertial stretching and mechanical break-off of solid or solidified part of shell forming a bump.

In this work, we study dynamics of a gold film of 40–100 nm thick on a silica substrate triggered by fast heating with ultrashort laser pulse. A sequence of involved physical processes leading to the formation of nanobump is discussed below. After several picoseconds of electron-ion relaxation, the pressure waves generated by such heating can result in spalling separation of film and its flying away from the substrate after the acoustic time *t*_*s*_=*d*_*f*_/*c*_*s*_ determined by the film thickness *d*_*f*_ and speed of sound in metal *c*_*s*_. Fluence of the heating laser beam in the plane of its cross-section can be represented by the spatial Gaussian distribution

(1) $$ F=F_{c}\exp\left(-r^{2}/{R_{L}^{2}}\right)  $$

where in-plane radius *r* is measured from the beam axis. Therefore, the heating of film surface is nonuniform along the cylindrical radius *r*. As a result of such heating, the initial (here it means after separation from a substrate) velocity distribution *v*(*r,t*=0) in material flying away from the substrate has a maximum *v*_*c*_(*t*=0)=*v*(*r*=0,*t*=0) at the beam axis. Thus, the separated film has the dome-like shape which inflates with time, and volume of an empty cavity between the separated film and the substrate increases during inflation.

Typical initial flight velocities are in the range of *v*_*c*_(0)∼30−200 m/s. The inflation stage can last from a few to several tens of nanoseconds if the diffraction-limited micrometer-sized laser focal spots (*R*_*L*_∼1 *μ*m) are considered. Capillary forces acting along the warped flying film decelerate inflation of a dome. Capillary deceleration of a bulging dome focuses mass flow along the dome shell in direction to its axis. This results in the formation of an axial jet and droplet on the top of the dome.

Here, our new simulation results and comparisons with experiments are presented. Those results in particular explain the formation of nanocrowns and appearance of debris in a form of frozen droplets lying on surface of irradiated spot. We demonstrate that the former is a consequence of break-off of the liquid part of the dome, and the latter is a result of the capillary return of droplet, respectively.

### Previous theoretical suggestions

**Fig. 1:**
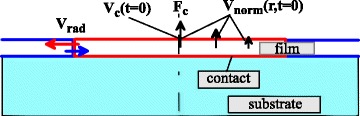
Hot disk (*red rectangle*) created by a laser action (1) with central fluence *F*_*c*_. *Black arrows* present the distribution of vertical velocity *v*_norm_(*r,t*=0) immediately after separation from a contact. See text about the meaning of the *red* and *blue arrows*

Main difficulty in understanding (solved recently in [22]), how the jet from the dome top appears, was around the focusing problem. It was obvious from the experiments with jetting that accumulation of mass of a heated spot takes place [17, 23, 24]. The material flows radially into the small central region forming a jet. This inflow causes strong thinning of a film outside to the central region. The thinning has been measured experimentally [24]. The scheme of experiments with a film/substrate target is shown in Fig. 1. A laser beam distributed according to (1) hits a target normally to its surface. The illumination produces a hot focal spot having a form of thin film disk marked out by the red rectangle in Fig. 1.

According to the previous suggestions, the hot disk expands laterally along a contact surface with velocities *v*_rad_ as a result of heating. This expansion motion collides with and reflects from a cold surrounding film indicated by blue color in Fig. 1. The expansion and reflection velocities correspond to the red and blue arrows in Fig. 1. The reflected wave propagates back to the center. Then, the cumulation of the reflected wave in the center strongly amplifies the wave. Collapse of the wave in the center produces vertical velocities. Thus, the central jet appears according to this consideration.

Amplification indeed can have a place in a cylinder thick enough. If we replace in Fig. 1 the thin red disk by the infinite vertical red cylinder placed into an infinite rigid blue tube, then amplification will happen, because the expansion flow induced by an initial pressure maximum at the axis can propagate outward, reflect from the rigid tube, and converge back to the axis. But for the thin films of interest, the aspect ratio *R*_*L*_/*d*_*f*_ is large, where *R*_*L*_∼1000 nm is a beam radius, *d*_*f*_∼40−100 nm is the thickness of a film. For such condition, the reflection is weak, and the pressure wave cannot propagate long distance through shallow liquid with a free boundary condition *p*=0 on the upper film boundary.

In our simple model, the initial (i.e., after film separation) radial velocities *v*_rad_, see Fig. 1, are small relative to velocities normal to the contact *v*_norm_/*v*_rad_∼*R*_*L*_/*d*_*f*_. Origin of the velocity field *v*_norm_(*r, t*=0) is connected with a vertical film/substrate hydrodynamic interaction through the underlying contact [22]; see Fig. 1. Velocity distribution *v*_norm_(*r, t*=0) mainly follows the fluence distribution (1) if the film/substrate adhesion is weak. It is known that a gold film used in experiments [17, 23, 24] is weakly coupled to a glass substrate. We have *v*_norm_(*r*,0)∝*F*(*r*) (1) everywhere inside the circle *r*<*r*_sep_ with the exception of the vicinity of ring *r*=*r*_sep_. Velocity *v*_norm_(*r*_sep_,0)=0 at the ring for the small but finite adhesion, while the distribution *F*(*r*) (1) exponentially damps out but remains non-zero.

Our model presented in [22] considers the following processes and phenomena: (i) two-temperature electron-ion relaxation stage, (ii) melting, (iii) film/substrate hydrodynamic interaction, (iv) formation of vertical velocity field (shown by the black arrows in Fig. 1) as a result of this interaction and separation of film from substrate (contact rupture) due to tensile stress coming to the contact with an acoustic wave, (v) early stage of inflation of bump *h*(*r,t*)=*v*_norm_(*r*,0) *t* following the initial vertical velocity distribution *v*_norm_(*r*,0), (vi) deceleration of vertical velocity *v*_norm_(*r,t*)<*v*_norm_(*r*,0) and focusing to the axis *v*_rad_(*r,t*)<0 of the liquid material, which forms a curved shell of bump as a consequence of the capillary forces acting tangentially along the curved surface, (vii) formation of an outward jet and an inward counter-jet, and decay of the outward jet into droplets.

Here, we omit discussion of above items and instead present development of the model based on Monte Carlo modeling of electron heat conductivity in material simulated by a classical molecular dynamics code. This allows us to take into account the strong electronic thermal transport in the case of metal and thus to describe both the spreading of heat along a film and the very fast cooling/crystallization of hot melt in the bump shell. The cooling solidifies molten metal and arrests the further destruction of a bump.

## Methods

### Classification of experiments and corresponding simulations

There is a list of experimental parameters: *F*_*c*_,*R*_*L*_ (they characterize a laser beam (1)), *d*_*f*_,*Z*_*f*_,*Z*_*s*_,*σ*,*χ*,*T*_*m*_ (they characterize a target), here *Z*_*f*_,*Z*_*s*_ are acoustic impedances *Z*=*ρc*_*s*_ of a film and a substrate, *σ*,*χ* are coefficients of surface tension and electron thermal diffusivity of metal *χ*=*κ*/*c*,*κ* and *c* are heat conduction and capacity, *T*_*m*_ is a melting temperature of metal. Let us create important combinations of these values to narrow the parametric space. The processes of capillary deceleration and focusing from the one side and the cooling and freezing from the other side are of primary significance. Therefore we introduce the capillary and thermal numbers

(2) $$ v_{0\sigma}= \frac{v_{c}(0)}{v_{\sigma}},\; v_{\sigma}=2\sqrt{\frac{\sigma}{\rho\, d_{f}}},\;\, v_{0\chi}=\frac{v_{c}(0)}{v_{\chi}},\; v_{\chi}=\frac{\chi}{r_{\text{sep}}},  $$

here *v*_*c*_(*t*=0) is velocity in the tip of a future bump at the instant of separation of a film from a contact, see Fig. 1, *ρ* is density of metal. The non-dimensional numbers *v*_0*σ*_,*v*_0*χ*_ (2) give strengths of the capillary and freezing effects relative to initial inflation velocity *v*_*c*_(0).

The films used in experiments are thinner than thickness of a heat-affected zone *d*_*T*_,*d*_*T*_≈140 nm for Au [25]. Thus, a film is uniformly heated along a normal direction at the stage of separation from a contact. This is the initial stage relative to the much later stage of deceleration of a bump. Initial increase of temperature is

(3) $$ \Delta T(r) = \frac{F(r)}{d_{f} \, c} - \frac{H_{m} }{3k_{B}} = 2.4\, \frac{F_{30}}{d_{50}}-0.5\, [\text{kK}],  $$

here *F*_30_=*F*/(30 mJ/cm^2^),*d*_50_=*d*_*f*_/(50 nm),*ΔT*(*r*)=*T*(*r,t*=0)−300, 300 K is room temperature of a target prior to laser action, *c*≈3*n*_0_*k*_*B*_ (Dulong-Petit law), *n*_0_ is concentration of atoms, *H*_*m*_=0.13 eV/atom is latent heat of melting of gold (enthalpy of fusion), *H*_*m*_/3*k*_*B*_=0.5 kK. Below a melting temperature *T*_*m*_, the second term *H*_*m*_/3*k*_*B*_ in (3) should be omitted. It is important that the pulse is ultrashort and that spread of absorbed heat from a skin layer is supersonic at the two-temperature stage [22, 26]: duration *t*_*T*_ of heating of a film in the normal direction is *shorter* than acoustic time scale *t*_*s*_=*d*_*f*_/*c*_*s*_. The latter defines the duration of mechanical film/substrate separation process. In these conditions, the pressure distribution created by a sudden temperature rise (3) is

(4) $$ p(r) \approx \Gamma \, c \, \Delta T(r) = 5 \, \Delta T(r) \, [\text{GPa}],  $$

here *Γ*≈2 is Gruneisen parameter and *ΔT* in [kK] is given by (3). This is an isochoric pressure increase.

Running acoustic waves are

$$p = \delta p \, f(x\pm c_{s} t), \;\; u = \mp (\delta p/Z) \, f(x\pm c_{s} t). $$

Expansion of heated (3) and suddenly pressurized (4) gold into the glass substrate produces a weak shock in glass and a rarefaction wave in gold. Pressure and velocity at the contact film/substrate are continuous, therefore

(5) $$ u_{cb}=\left(p-p_{cb}\right)/Z_{f}=p_{cb}/Z_{s}, \;\; p_{cb}=p/\left(Z_{f}/Z_{s} + 1\right),  $$

where *u*_*cb*_ and *p*_*cb*_ are values at the contact boundary and *p* is given by expression (4). Pressure *p*_*cb*_ (5) is positive, the contact moves in a substrate direction. This continues up to the instant 2*t*_*s*_ when a wave sent from a contact at *t*≈0 will reflect from a vacuum boundary of a film and will return back to the contact. Pressure [GPa], velocity [m/s], and shift [nm] of a contact during expansion are

(6) $$ p_{cb}=0.6 \, \Delta T,\;\; u_{cb}=70 \, \Delta T,\;\; 2 \, t_{s} \, u_{cb} = 2\, d_{50} \, \Delta T.  $$

We take values *ρ*_*f*_=19.3 g/cm ^3^,*ρ*_*s*_=2.2 g/cm ^3^,*c*_*s*_|_film_=3.3 km/s, *c*_*s*_|_substr_=3.9 km/s for gold (f) and glass (s) when we calculate impedances and coefficients in these expressions.

At the instant *t*=2*t*_*s*_, the expansion half-period 0<*t*<2*t*_*s*_ finishes and the contraction half-period begins. In the linear acoustic approximation (valid if pressures are much less than a bulk module and motion is subsonic) the contact pressure *p*_*cb*_ and velocity *u*_*cb*_ given in (6) change their sign to the opposite sign during the contraction half-period. If contact adhesion is weak then a contact break-off at the beginning of a tensile stage, i.e., at the beginning of the second half-period, is done. In this case, a momentum of a film accumulated during the first half-period defines separation velocity *v*=2*t*_*s*_*p*_*cb*_/(*ρd*_*f*_)=2*p*_*cb*_/*Z*_*f*_=19 *ΔT* [m/s] with *ΔT* from (3); compare velocities *v*=2*p*_*cb*_/*Z*_*f*_ and *u*_*cb*_=*p*_*cb*_/*Z*_*s*_ (5), *v*≈*v*_*cb*_/3.7 for the gold/glass film/substrate target. Comparing this approximate expression for *v* with the two-temperature hydrodynamics results, we see that the expression for *v* underestimates separation velocity ≈2 times. This follows from the table presented in [22], p. 24. The corrected expression for the separation velocity is

(7) $$ v \approx 38\, \Delta T \, [\text{m/s}].  $$

Using velocity (7) as *v*_*c*_(0), we obtain values for the non-dimensional parameters (2). For the capillary number, we have

(8) $$ v_{0\sigma} = 0.6 \, \sqrt{d_{50}} \, \Delta T,  $$

where it is supposed that *σ*=1000 dyne/cm.

Significant vertical displacement of a film shown in Fig. 1 begins in the point *r*_*m*_ where the film is molten. Therefore, we suggest that the radius of appreciable deviation of a film from substrate is *r*_*m*_ and we will regard below this radius as the separation radius *r*_sep_=*r*_*m*_. For gold (*T*_*m*_=1.337 kK), the melting threshold on absorbed fluence according to (3) is

(9) $$ F_{m} = 19 \, d_{50}\, \left[\mathrm{mJ/cm}^{2}\right],  $$

compare with table composed for *d*_*f*_=60 nm gold in [22]. Expression

(10) $$ r_{m} = R_{L} \sqrt{\ln F_{c}/F_{m}} = R_{L} \sqrt{\ln \left[(F_{c})_{30}/\left(0.64\, d_{50}\right)\right]}  $$

follows from the distribution (1) and formula (9).

Let us calculate the thermal number *v*_0*χ*_=*v*_*c*_(0)/*v*_*χ*_,*v*_*χ*_=*χ*/*r*_*m*_ (2). From Eqs. (7) and (10), we have

(11) $$ v_{0\chi}= 0.76 \,\, \Delta T \, R_{\mu} \, \sqrt{\ln \left[(F_{c})_{30}/\left(0.64\, d_{50}\right)\right]}.  $$

A beam radius *R*_*L*_ (1) is *R*_*μ*_=*R*_*L*_/(1 [*μ*m]) in non-dimensional expression (11); we take *χ*≈0.5 cm ^2^/s for molten gold to calculate *v*_*χ*_.

**Fig. 2:**
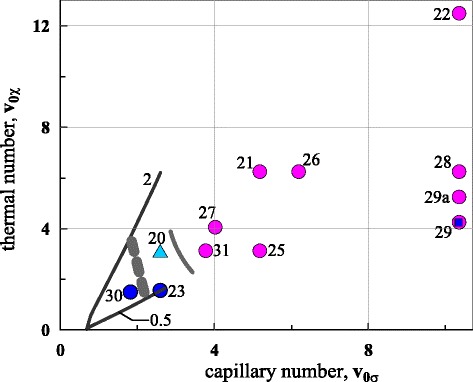
The curves 0.5 (*R*_*L*_=0.5 *μ*m) and 2 (*R*_*L*_=2 *μ*m) present estimates of location of experimental parameters for a gold film *d*_*f*_=60 nm on a glass substrate. Typically, they cover the range 2*F*_*m*_<*F*_*c*_<3*F*_*m*_ around the *dashed grey curve* separating the experimental non-ejecting (they are closer to the origin point) and ejecting regimes. The circles shows the simulation runs. The grey continuous curve separates non-ejecting (*the blue circles*) and ejecting cases (*the red circles*). We see that the simulations rather satisfactory correspond to the experiments

The map of numbers *v*_0*σ*_,*v*_0*χ*_ is presented in Fig. 2. The curves 0.5 and 2 shows parametric dependence *v*_0*σ*_(*F*_*c*_,*d*_*f*_=60 [nm]) (8) and *v*_0*χ*_(*F*_*c*_,*d*_*f*_=60 [nm],*R*_*L*_) (11) as function of the central fluence *F*_*c*_ as a running parameter varying in the range *F*_*m*_<*F*_*c*_<3*F*_*m*_ for the radii *R*_*L*_ equal 0.5 *μ*m (curve 0.5) and 2 *μ*m (curve 2). If *F*_*c*_=*F*_*m*_, then *r*_sep_=*r*_*m*_=0. In this case, the thermal velocity *v*_*χ*_=*χ*/*r*_*m*_ is infinitely large, while thermal number *v*_0*χ*_=*v*_*c*_(0)/*v*_*χ*_ is zero. Therefore, the curves 0.5 and 2 in Fig. 2 start from the horizontal axis. The hole in a film appears for the higher fluences *F*_*c*_ > 3*F*_*m*_ and regime of separation can change because internal spallation of a film begins.

Main new achievement used in the paper here (relative to the previous work [22]) is addition of the Monte-Carlo (MC) algorithm to the molecular dynamics (MD) algorithm [27]. The sense of the MC modeling is the following. The MC electron jumps from its host atom to the neighbor of the host thus transporting heat from hot to cold place along the electron subsystem. The quantity *ν*_*D*_ gives frequency of these jumps per electron. The rate of electron-ion kinetic energy exchange is defined by two parameters: the second frequency *ν*_ei_ and effective mass of electron *m*_ef_. The value *ν*_ei_ is electron-ion collisional frequency. Higher frequencies and mass *m*_ef_ correspond to the stronger electron-ion thermal coupling. We achieve the necessary value for the thermal diffusion *χ* varying the triple of parameters *ν*_*D*_,*ν*_ei_, and *m*_ef_. The coefficients *χ* used in simulations are listed in Table 1.

**Table 1:** Parameters of the MD-MC simulation runs

#	*N*_atom_/10^6^	*χ* cm ^2^/s	*L*_box_	*d*_*f*_	*v*_0*σ*_	*v*_0*χ*_
20	6.0	0.09	140	5.1	1.3	1.6
23	6.0	0.17	140	5.1	2.6	1.6
30	44	0.17	270	10	1.9	3
3	192	0	620	8.3	2.6	*∞*
21	6.0	0.09	140	5.1	5.2	6.3
22	6.0	0.09	140	5.1	10	13
25	6.0	0.17	140	5.1	5.2	3.2
26	2.1	0.17	140	1.8	6.2	6.3
27	2.1	0.17	140	1.8	4	4.1
28	6.0	0.17	140	5.1	10	6.3
29	6.0	0.17	140	5.1	10	4.3
29a	6.0	0.17	140	5.1	10	5.3
31	44	0.17	270	10	3.8	3.2

Number of millions of atoms is given in the *second column*. Monte Carlo (MC) algorithm allows to vary electron heat conduction *κ* and hence the heat diffusion coefficient *χ*. We adjust *χ* to correspond to the experimental values of *v*_0*σ*_ and *v*_0*χ*_ (see Fig. 2), while having smaller spatial scales in a simulation. The *rectangle* of the simulation box has a square cross-section *L*_box_×*L*_box_ in the plane of a target. Values *L*_box_ and *d*_*f*_ are given in [nm]

Decreasing diffusivity *χ* in comparison with an experimental value, we create smaller numbers of atoms *N*_atom_ in computational system approximately equivalent to the real experimental system. This is equivalently relative to the capillary and thermal numbers (2). Mechanical and thermal dynamics of the real and the simulated objects should be similar if their numbers (2) are equal because the inertia/capillary and inertia/freezing processes govern dynamics. Parameters of the runs simulated by the molecular dynamics combined with Monte Carlo (MD-MC) program are listed in Table 1 and in Fig. 2. Numbers near the circles in Fig. 2 correspond to the numbers (#) of the runs in the first column of Table 1. We see that there is approximate correspondence between the experimental and simulation regions in Fig. 2 if we exclude the runs with the highest velocities *v*_*c*_(0).

As was said, the experimental and simulated objects are approximately equivalent. At the same time, the number of atoms *N*_atom_ in simulation is hundred times less. The simulations shed light onto the internal processes running inside the illumined hot-spot invisible experimentally.

## Results and discussion

### Solidification of bump, jet formation, separation of droplets, and destruction of bump

The idea of miniaturization of the MD-MC runs using *v*_0*σ*_,*v*_0*χ*_ parameters (2) has been presented above. Experimental (the curves 0.5, 2) and simulation (the circles) situations on the map of these parameters are shown in Fig. 2. The curves 0.5, 2 have been obtained from the estimates (8) and (11). In experiments, the bumps appear at *F*_*c*_∼2*F*_*m*_. The jets are formed at *F*_*c*_∼2.5*F*_*m*_. Somewhere above 3*F*_*m*_, ejection and destruction begin. The thick dashed curve in Fig. 2 marks the approximate position of the line separating non-ejecting and ejecting regimes in experiments. At the present, in experimental papers, the values of energy of pulse are given. It is difficult to obtain precise values for the absorbed central fluence *F*_*c*_ from these energies; *F*_*c*_ is necessary for (8), (11). Using simulation results, let us consider how dynamics and the final shapes change with variation of position of the point on the map of the parameters in Fig. 2.

**Fig. 3:**
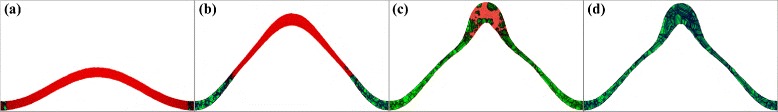
Evolution of the shape and gradual freezing of molten gold are shown. This is the run # 30 from Table 1 where the sizes are given. *Green* is solid gold, while *red* is liquid. Liquid is strongly overcooled below the melting point. Thus, nanocrystallites appear not only at the boundary of continuous solid with the liquid part but also inside the liquid part. The cross-section of the bump is presented. Thickness of this cross-section is 3 nm in direction normal to the plane of picture. The symmetry parameter shown by the *green/red colors* (defining the solid/liquid states) is obtained by averaging along this 3 nm. **a** *t*=216 ps, **b** 0.72 ns, **c** 1.37 ns, **d** 2.34 ns

Blue symbols in Fig. 2 correspond to the non-ejecting cases. They are the low-velocity cases, when the rate of freezing is enough to crystallize the bump as whole before its destruction. Hydrodynamic velocity and hence influence of inertia increase as we go farther from the origin in Fig. 2. The jet begins to form in the run # 30 shown in Fig. 3. But kinetic energy is relatively low, and the capillary and freezing effects are stronger; thus, only the embryo of the jet had time to develop. The colors show a distribution of the symmetry parameter: the more green colors correspond to the more ordered structures, green is a crystalline state while red is liquid gold. The run # 23 marked in Fig. 2 behaves similar to the run # 30 in Fig. 3; again the underdeveloped jet is formed in the tip of the bump.

**Fig. 4:**
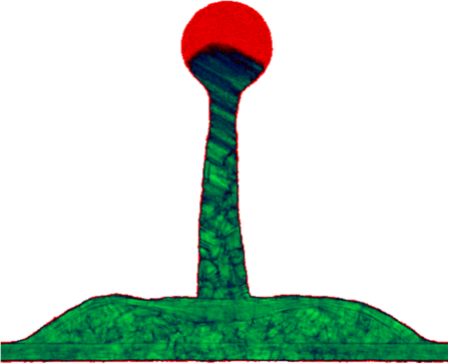
This is the run # 20 shown in Table 1. It is located near to the grey continuous line separating the simulated non-ejecting and ejecting cases in Fig. 2. The instant *t*=2.3 ns is presented. Solid parts are painted by *green color* and liquid parts by *red color*. The figure is obtained by summing and averaging the symmetry parameter along a normal direction perpendicular to the plane of the figure. Therefore, the *thin horizontal line* (slightly above the bottom) appears; it marks the pedestal formed by the gold film remaining in contact on a surface of a substrate. The same pedestal is clearly seen in Fig. 7 below. Also, the *thin curves* near the upper boundary of the shell of the bump show the internal boundary of the shell from the side of the internal empty cavity. Let us mention that the solidified shell undergoes few damping oscillations up and down before it transits into a rest state

The longer (relative to the # 30 in Fig. 3) jet develops in the case # 20 presented in Fig. 4. This is the case with significantly larger initial kinetic energy (see Fig. 2) in comparison with the run in Fig. 3. The case # 20 is near the separation line bounding the non-ejecting cases: the long jet and the droplet ready for separation are grown enough. The freezing only slightly advances the detachment of the droplet. The approximate position of the separation or bounding line for the MD-MC simulations is given in Fig. 2 by the continuous grey curve separating the blue and reddish symbols. Snapshot of material states, including red-colored melt and green-colored solid, at time *t*=2.3 ns is shown in Fig. 4. Approximately 40 % of the droplet is frozen to this moment. The simulation # 20 is continued up to the final frame (not presented here) corresponding to the time *t*=2.7 ns, when the droplet is solidified entirely.

The mechanics of formation of the droplet at the end of a liquid jet or a thickened rim around the hole in a liquid membrane has been considered in [22] in the case of a bump and in [28] in the case of membranes in a foam; see also Figs. 8 and 9 below. If we cross the jet perpendicularly by two slightly separated planes, then there are two closed contours belonging to the crossed liquid surface of a jet. There are capillary forces acting on to the cutting of a jet between these two planes. These forces act on the first and the second contours and compensate each other if the lengths of the contours are equal; if the lengths are not equal, then this effect (together with a force acting perpendicular to a jet) favors the compression of the more narrow part of a jet during the Rayleigh instability. Let us now cut the neck of the droplet by the same plane. We see that the compensating forces of surface tension are absent. Therefore, the tension existing in the neck decelerates the droplet relative to the neighbor part of the jet. The droplet rakes in mass from the jet. This is the reason why a droplet or a rim is formed. In [22], calculation of corresponding velocities are given. It is surprising that these velocities are moderate in the sense that the shell of a bump has time to expand even if there is a hole in a shell. It is surprising because it seems that the whole shell or membrane is in strongly stressed state and thus the area of the membrane should contract immediately after the appearance of a hole. Indeed, it contracts but not immediately.

**Fig. 5:**
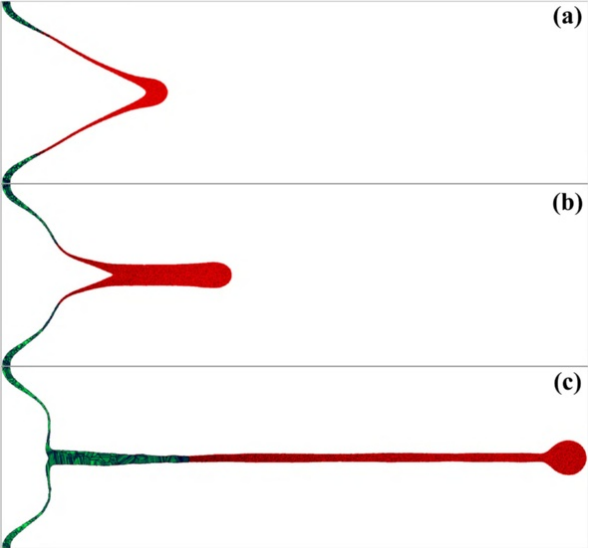
Development of the very long jet in the case # 31 presented in Table 1 and in Fig. 2 is shown. The instants are (**a**) *t*=0.72 ns, (**b**) 1.15 ns, (**c**) 4.536 ns. Solid is *green*; liquid is *red*. Significant redistribution of mass from the shell of a bump to the jet takes place. We see that the shell in its middle part and the part near the jet is much thinner than in the part near the substrate where the shell keeps its initial thickness. We also see the flattened vicinity of the tip of the shell of the bump, compare with previous Fig. 4. Such flattened tips are often observed in experiments, e.g., see Figure 2c in [17] and Figure 4c in [23]

The run # 31 presented in Fig. 5 is slightly above the grey line in Fig. 2 isolating the non-ejecting runs (the blue symbols). Much longer jet develops in the run # 31 in Fig. 5 relative to the run # 20 in Fig. 4. As we wait for a long time, the large droplet is gradually formed at the end of the jet while the jet becomes thinner and thinner—compare the frames (b) *t*=1.152 ns and (c) *t*=4.536 ns. At the same time, the crystallization front moving along the jet is still far from the droplet, compare with Fig. 4. It seems that all is ready for separation of the first large and fast droplet. May be smaller and slower droplets will accompany the large one. If we suppose that the hardly probable case of full freezing of the jet in Fig. 5c will nevertheless takes place, then this will contradict to the experimental observations [1, 7, 9, 17, 23, 24] where never such large ratios of a length of a jet to a bump separation radius *r*_sep_ have been obtained.

Let us also compare the positions of the points corresponding to the runs # 30 and # 31 on the map in Fig. 2 and propagation of the green solidified area in Fig. 3c, d and in Fig. 5a. In the low-velocity case # 30 and in the higher velocity case # 31, the shapes of the bumps are similar (this is work of inertia and surface tension) while the solidified parts are very different. This is the relative delay of solidification in the faster case # 31.

**Fig. 6:**
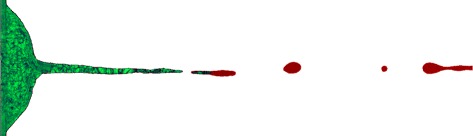
Formation of the solidified bump, the peaked solid (*green*) jet, and the sequence of flying liquid (*red*) droplets in the run # 25. The instant is *t*=2.232 ns. We see that increase of the capillary number *v*_0*σ*_ relative to the case # 31 (compare the points # 31 and # 25 in Fig. 2) leads to decay into droplets at the stage when the jet # 31 is still continuous, compare with Figs. 5 and 6. They are spread in sizes of the droplets. The last one separates semisolid

Additional increase of velocity *v*_*c*_(0) above the value corresponding to the run # 31 in Fig. 5 causes faster decay of a jet into the train of successive droplets. This is the example # 25 shown in Fig. 6. They are spread in velocities, temperatures, and sizes of the droplets. The first droplets move faster and are hotter. The last droplet, which recently separates in the instant shown in Fig. 6, is semisolid. The liquid part of this droplet is overcooled below the melting temperature. Thus, this droplet will crystalize soon. The first droplets can fly in a liquid state for a long time because the rate of radiative cooling is extremely low. The angular distribution of the droplets in their cloud is very narrow if they are produced in the process of decay of a long jet. At even higher kinetic energies, the part of a shell can decay into cloud of droplets, then the angular distribution of droplets is wider.

**Fig. 7:**
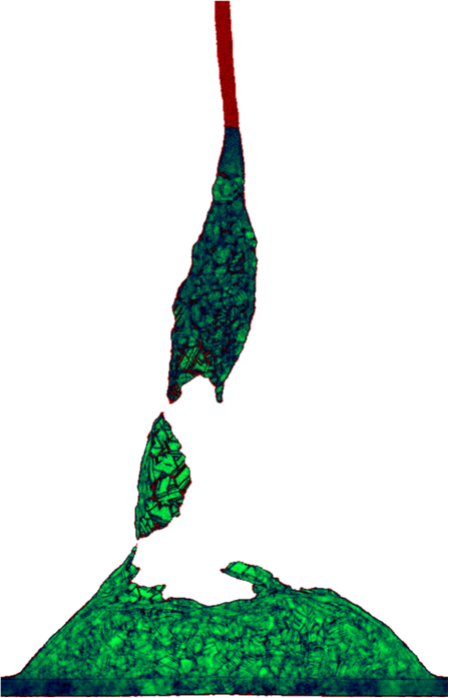
The run # 29 with the largest initial velocity *v*_*c*_(0) together with the fastest crystallization rate; see Fig. 2 and text for explanations. The instant *t*=0.729 ns is shown. Large velocity *v*_*c*_(0) and fast cooling result in the rupture of the shell in its solidified state. Only the bottom part of a superlong jet is shown. The jet decays into many liquid droplets

The runs ## 22, 28, 29a, and 29 have the highest velocity values *v*_*c*_(0) investigated in our runs listed in Table 1 and in Fig. 2. Corresponding capillary numbers *v*_0*σ*_ are the same for these runs because a coefficient of surface tension is the same for all runs. Let us discuss the thermal numbers since their values are important for results. To cool a bump in the MD-MC runs, we use a Langevin thermostat. First, a molten film has been prepared by increase of temperature above the melting point. The temperature profile supported by the thermostat has the maximum temperature 2000 K in the center. Temperature decreases down to 1500 K in the periphery of the simulation box. We switch off the thermostat inside the circle *r*<*R*_sep_ after creation of an equilibrium liquid. While in the periphery, we steadily use the thermostat (i) to keep a film at the periphery in contact with the substrate (therefore, the pedestals in Figs. 4 and 7 are in rest at the substrate) and (ii) to support temperature at the periphery below the melting temperature. This thermal condition corresponds to real situation where outside the molten hot-spot a film is solid at room temperature. The supported temperature is 500 K for all runs except the runs # 29a and # 29 where it is 300 K. The run # 22 has small thermal velocity *v*_0*χ*_, see Table 1, relative to the cases ## 28, 29a, and 29. The runs # 29a and # 29 are below the run # 28 because the supported temperature is lower (300 K versus 500 K). The characteristic time of thermostat is 0.8 ps for all runs except the run # 29a where it is 1.2 ps. Therefore, cooling in the case # 29a is slower than in the case # 29. The circles in Fig. 2 are plotted according to their capillary and thermal numbers (2). Only the circles # 29a and # 29 are shifted down relative to the point # 28 in accordance with the estimates following from the differences in the temperature at a periphery and the characteristic time of thermostat.

Thus, the run # 29 has the largest kinetic energy and the fastest cooling. It is singled out by the blue square inside the red circle in Fig. 2. Figure 7 shows instant 792 ps of the run # 29. The run is significantly above the non-ejecting limit (the grey continuous line in Fig. 2). An extremely long jet is produced. Only a small part of the jet is presented in Fig. 7. The jet decays into multiple small droplets not visible in the truncated Fig. 7. The crystallization front moves fast enough to solidify the whole shell and the bottom part of the jet when there is still a significant stretching velocity gradient *du*/*ds* along a solid part; *s* here is length of an arc, and *u* is the tangential component of velocity. The inertial forces connected with this gradient breaks off the shell. Strong thinning of a shell does easier the break off. The thinning of a shell results from transfer of large amount of mass into the jet; see Fig. 5 above. By this means a large slowly moving piece of a solid shell may appear in the ejecta in addition to the droplet component.

#### Self-developed inhomogeneities and break through of shell

Initial data for a MD-MC simulation are smooth. A run starts from an ideal single fcc gold crystal with the exact plane boundaries. After that, the heating and melting by a thermostat take place and a smooth (analytical function) vertical (see Fig. 1) velocity profile is imposed. Therefore later, only the thermal fluctuations, possibly in combination with some kind of hydrodynamic instability, cause deviations from ideal smoothness—there are no any initial perturbations. Instability may be the modified Rayleigh-Taylor type one, but deceleration, necessary for this type, is caused by surface tension acting on the largest scale ∼*r*_sep_. Thus, it is relatively weak to drive the much shorter than *r*_sep_ wavelengths, because for them the surface tension plays stabilizing role overcoming destabilizing deceleration since wavelengths are much shorter than *r*_sep_. But this conclusion is valid for the plane thin membrane when we compare the long and short wavelengths with the infinitesimally small amplitudes. It is unknown how the situation will change when the largest wavelength has a finite amplitude and/or the perturbation wavelength is less than *r*_sep_ but is not much smaller. Theoretical study of a thermal excitation of a capillary wave spectrum and instabilities needs a separate work. Here, we present results exploring violation of smoothness in MD-MC simulations.

**Fig. 8:**
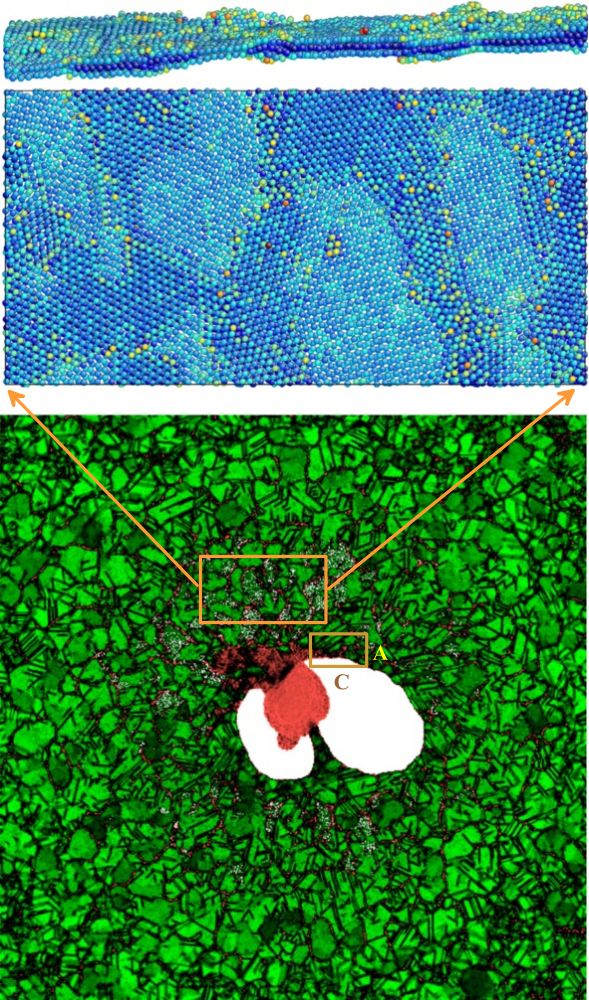
*Bottom.* The top view onto the shell of a bump in the run # 27 at the instant *t*=0.475 ns. The *square* is the cross-section of the computational box in a plane of a substrate. A distribution of a symmetry parameter defining an interatomic order is given. The parameter changes in the range from *green* (fcc lattice) color to *red* (molten gold, the central jet). The *yellow rectangle* marks the area of the shell for which the AtomEye (atomistic configuration viewer) middle and upper views in this figure are shown. An atomistic representation of the second rectangular (A,C) is given in Fig. 9. *Middle.* The top view onto the atomic ordering. In the middle and the upper panels, the individual atoms are colorized according to their potential energy—*deep blue* corresponds to the strongest binding, *less blue* colors mark weaker binding—these atoms are higher at the axis of potential energy. *Top.* The lateral AtomEye view onto the same piece of the shell. The lateral cross-section shown in the foreground is the upper horizontal side of the middle rectangle, compare correspondence of colors

The top view onto the frozen shell and the flying up (relative to the plane of the bottom panel in Fig. 8) jet is shown in the bottom of the three panels in Fig. 8. The jet at this instant has a height 2.3*L*_box_ and remains molten; *L*_box_ is the length of the computational cross-section. To imagine the jet, see Figs. 5, 6, and 7 with the similar jet structures. We clearly see the net of domains (nanocrystallites) grown from the seed crystallization grains; see also Figs. 3, 4, and 7. The liquid is overcooled, thus the isolated grains appear in the transition solid-liquid zone; see Fig. 3. Figure 3 presents distribution of an averaged (along line of view) symmetry parameter in a thin vertical crossing while in Figs. 4 and 7 the averaging is made along a line of view intersecting thicker pieces of matter. They are especially thick in the lateral regions where the line of view passes matter tangentially to the shell. Thus in these regions, the net of the crystallites is smeared due to averaging, compare with Figs. 3 and 8 (bottom) where the smearing is absent.

**Fig. 9:**
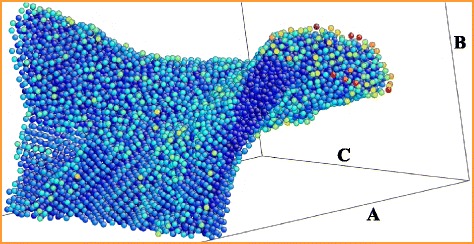
Cumulation of mass in the rim around the hole according to the AtomEye view; again, atoms are colored by their potential energy. The cumulation is a result of raking of material particles forming a shell in a process of expansion of a hole, compare with cumulation of mass into the head droplet in Fig. 4. The particles having their instant positions outside relative to the rim do not “know” about the hole and about its expansion at this particular instant [22]. The *rectangular box* A-B-C has the basement A-C corresponding to the rectangle A,C in Fig. 8 (*bottom*). The thickening corresponds to the rim located around the boundary of the hole crossed by the rectangle A,C in Fig. 8

Figures 8 and 9 are designed to emphasize the pattern of compactions/depressions. The pattern is formed from capillary oscillations [29, 30] during the stage when the shell was liquid. Rapid crystallization fixes the pattern. The reasons for appearance of the compactions in the map in Fig. 8 in the internal rectangle and in the rectangle A,C in Figs. 8 (bottom) and 9 are qualitatively different. In the internal rectangle, we see the random compactions; they are the remnants of the thermally induced capillary waviness [29, 30] (may be in combination with a hydroinstability). While in the rectangle A,C the main compaction is regular (the rim), it is caused by raking of mass by the expanding hole. The hole expands when a shell is liquid. Freezing fixes the rim.

The deep blue atoms in Fig. 9 are the bulk atoms distantly located from the two surface monolayers. We see them in the cut made by the plane A-B. These atoms form the thickening called the rim. The plane A-B intersects the rim at an angle. The atoms at the edge surface of the rim are yellow-red colorized. We see also the spot of depression located at the left side in Fig. 9. Inside the depression, the visual lines pass through the empty gaps between atoms. There are only two monolayers remaining in the depression; therefore, the gaps are seen.

We pay attention to the chaotic net of the alternating compactions and depressions because namely progression of the depressions leads to perforation of a shell. The spots of depressions and the stripes of compaction are seen in Fig. 8 (bottom). The depressions correspond to the greyish spots. They look like perforated. The inspection of the random pattern is presented in the top and middle panels in Fig. 8. The inspection is made by the Linux-based software AtomEye capable of visualizing atomistic configurations. This software allows us to see better the inhomogeneous structure.

In Fig. 8, the individual atoms are colorized in correspondence with potential energy. In the more blue regions, the shell is thicker (this is compaction or condensation); there are the bulk; most blue atoms are there. There are no gaps in the condensations. While we see the empty small gaps between the atoms in the depressions in Fig. 8 (middle), where only two surface monoatomic layers remain. Figure 8 (top) clearly demonstrates that indeed there are only two monolayers in the thinnest spots of a shell. There are pairs of atoms in these spots (one above another) having light blue color indicating that potential energy is small. On the other hand, there are deep blue colors in the thicker spots where 3–4 monolayers are present.

## Conclusions

Production of solitary surface nanostructures by the ultrashort laser pulses is used in many applications listed in the “Background” section. Productive process is mainly governed by the interplay between inertia, capillarity, and solidification. Therefore, it can be described on the two-dimensional plane of the capillary and thermal numbers. In the paper, the regimes corresponding to the experimental set (parameterized in experiments by energy of an incident pulse within a fixed focal radius) are studied. The regimes change with energy increases. We have described dynamics in these regimes starting from a simple bump, to a bump with a nipple (## 23, 30, Fig. 3), after that to a bump with a frozen jet (near the limit of non-ejecting cases, Fig. 4), and after that the ejecting regimes begin (Figs. 5, 6, and 7).

Description is based on a combination of numerical codes and physical approaches. Physical picture of inflation, stopping, and crystallization of bump includes the thermal and mechanical transport of heat and momentum from the hot shell to the external cold part of a film remaining in contact with substrate outside the bump. The combination of codes includes two-temperature hydrodynamics (2T-HD) code together with MD-MC code (molecular dynamics combined with Monte Carlo method). We run series of the 2T-HD simulations to explore dependence of separation velocity of gold film and its temperature on the absorbed fluence. This part of work is described in [22, 31]. New results presented above are based on the usage of the Monte Carlo code for a heat transport problem for implementing the fast freezing of the bumps with material molten by an ultrashort laser heating. Formation of chaotic solidified inhomogeneities is described in Figs. 8 and 9.

Similarity on the capillary *v*_0*σ*_ and thermal *v*_0*χ*_ numbers allows us to use scaling in MD-MC simulation of a large number of involved atoms *N*_atom_ in experimental system by a smaller atomic system. Typical experimental sizes vary from the beam radii *R*_*L*_ of the order of few micrometer to the submicrometer radii in the cases where the diffraction-limited tight focusing UV lasers or higher harmonics of a Ti:sapp laser are used. With the scaling, we correctly describe formations of a bump, short and long frozen jets, and production of droplets by fragmentation of the long jets. Thresholds between these regimes agree satisfactorily with estimates of the experimental thresholds; see Fig. 2. Here it is worth to note that, unfortunately, accurate estimates of absorbed fluence *F*_*c*_ are not available in the experiments.

Again, the scaling equivalence is exact for the listed processes: formation of bumps, jets, and droplets from jets. Indeed, even a complicated process of the droplet production is governed by the surface tension and the exponentially fast Rayleigh instability. Thus, it is enough for a jet radius in simulations to be larger than few interatomic distances. This demand is hold in our simulations. In this connection, it is necessary to say that a phenomenon of restoration of the surface tension coefficient with a number of interatomic layers is carefully studied; see Figs. 8 and 9 and Appendix in [22]. We are not sure only in accuracy of our description of the process of formation of a hole in a stretched film, Figs. 7, 8, and 9. It seems that our simulations here give the order-of-magnitude estimates of timing and threshold. It is necessary to stretch a film down to 1-nm thickness to cause a rupture. In simulations, we use from few times to one order of magnitude initially thinner films. It is not clear if exponentially fast instability presented here determines the duration of rupture by its inverse increment (like the Rayleigh instability of cylindrical jets). If not, then the duration depends on initial thickness and will be faster for thin films. There are many cases where very strong thinning of films and formation of holes are observed in experiments.

In conclusion, we can say that our model successively describes several phenomena. Among them are

- deceleration and solidification of separated film;
- appearance of the conical bumps (Fig. 3);
- formation of the short tip on the top of bump (Fig. 3);
- the moderately long jet with the head droplet (Fig. 4);
- formation of the flattened vicinity in the shape of bump; it is located around a jet (Figs. 4 and 5);
- formation of the extremely long jets (Figs. 5 and 6) and very strong thinning of the bump shell (Fig. 5);
- appearance of the droplets (Fig. 6);
- the hypothesis about possible presence of the solid fragments of shell and/or jet in ejecta (Figs. 6 and 7);
- the study of development and freezing of the chaotic (Fig. 8) and regular (the rim, Fig. 9) inhomogeneities formed from smooth initial data.
